# Intrasheath Tendon Subluxation Beyond the Ankle: The First Case Series in the Hand

**DOI:** 10.1002/jum.70020

**Published:** 2025-07-31

**Authors:** Hannah Lamberg, Molly N. Pantelic, Erika Mojica, Heidi Ehrich, Dana Rowland, Shannon Wood, Gunjan Malhotra, Steven B. Soliman

**Affiliations:** ^1^ Division of Musculoskeletal Radiology, Department of Radiology University of Michigan Ann Arbor Michigan USA

**Keywords:** dynamic ultrasound, extensor compartments, hand, intrasheath subluxation, musculoskeletal ultrasound, tenosynovitis

## Abstract

Peroneal tendon intrasheath subluxation is a well‐established phenomenon in musculoskeletal ultrasound. However, until now, intrasheath tendon subluxation has not been described in the hand. This unique case series presents the first three reported cases of intrasheath tendon subluxation in the hand. We discuss the anatomy of the flexor and extensor tendons, highlight dynamic sonographic findings, and propose possible mechanisms for this entity. Outside of the ankle, intrasheath subluxation is an uncommon finding, infrequently considered, and easily overlooked, particularly without the use of dynamic ultrasound. Knowledge of this entity and its sonographic features is crucial for its accurate diagnosis.

**Abbreviations**
APL: abductor pollicis longus
EPB: extensor pollicis brevis
FDP: flexor digitorum profundus
FDS: flexor digitorum superficialis
US: ultrasound

Intrasheath subluxation of the peroneal tendons in the ankle, characterized by an abnormal relationship between the peroneus longus and brevis tendon locations during movement, is a well‐established phenomenon in musculoskeletal ultrasound (US).^1^, ^2^, ^3^, ^4^ However, this condition has not been reported in other extremities until now. We present, for the first time, a unique series of three cases detailing intrasheath subluxation in the hand. These cases include a review of clinical history, dynamic US techniques, sonographic findings, and patient management. Intrasheath subluxation outside of the ankle is an uncommon finding, infrequently considered, and easily overlooked, particularly in the absence of dynamic ultrasound. Knowledge of this entity and its sonographic features is crucial for its accurate diagnosis.

## Materials and Methods

This study was performed in accordance with the ethical standards of our institutional research committee and with the 1964 Helsinki declaration and its later amendments or comparable ethical standards. Institutional review board protocol review and informed consent were exempt per our institutional review board policies for this type of manuscript and since these examinations were clinically indicated. Our study complied with the Health Insurance Portability and Accountability Act.

All US examinations were performed by trained dedicated musculoskeletal sonographers, all of whom possess the registered MSK sonographer (RMSKS) designation through the American Registry for Diagnostic Medical Sonography (Rockville, MD, USA). For each patient, the US was performed utilizing an 18‐MHz compact linear transducer (GE LOGIQ E9 unit; General Electric Company, Milwaukee, WI, USA).

## Results

### Case 1

A 28‐year‐old right hand dominant woman was evaluated in an outpatient orthopedics clinic due to a 4‐year history of right wrist and hand symptoms. She specifically reported an intermittent painful popping and locking sensation at the radial aspect of the right wrist, occurring approximately once per week and resolving after shaking the affected hand. She also reported decreased range of motion in the right thumb compared to the left. Radiographs were normal. A musculoskeletal US was requested to evaluate the dorsal wrist with attention to the first and second dorsal extensor compartments.

**Figure 1:**
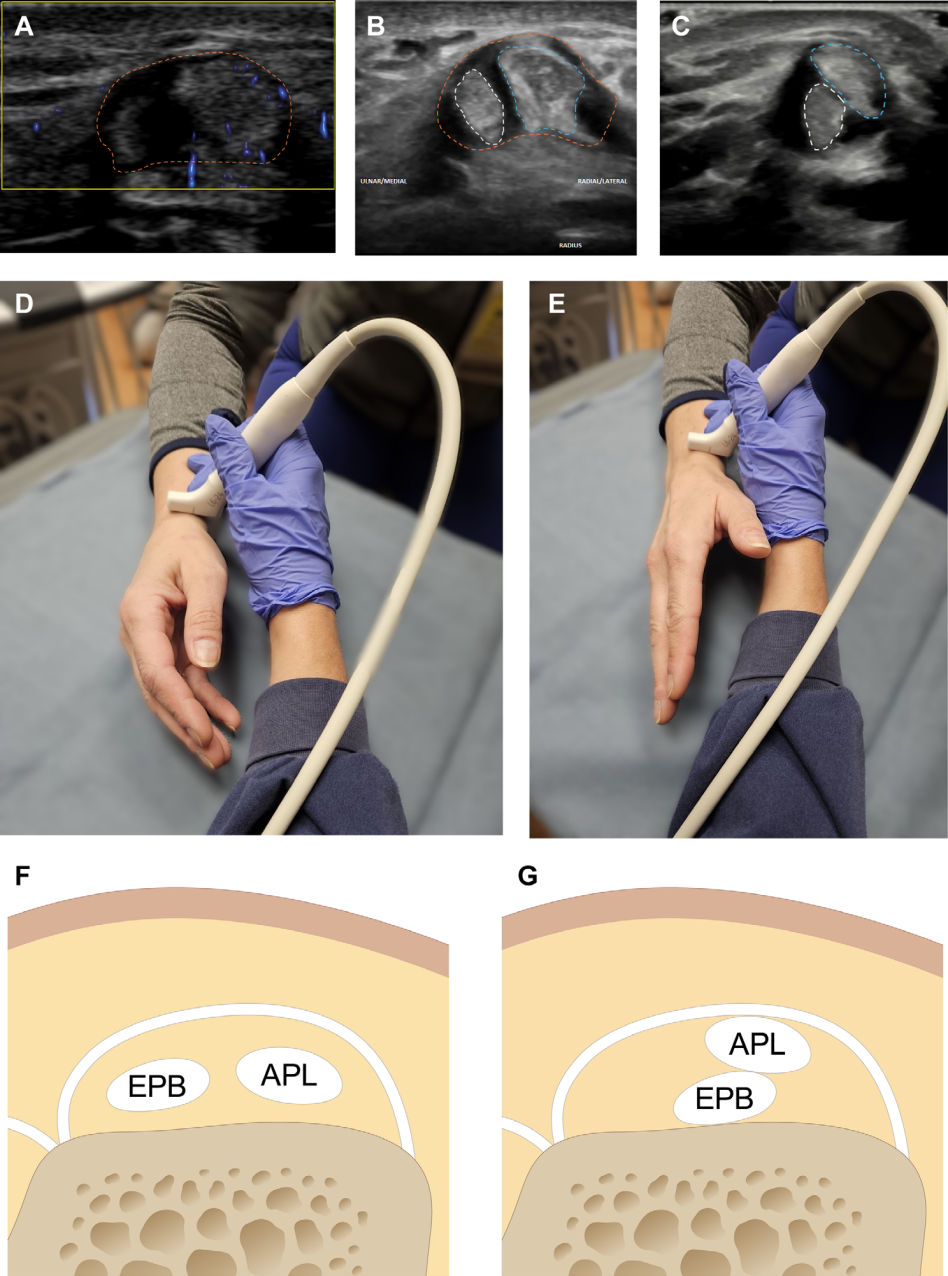
De Quervain tenosynovitis in a 28‐year‐old woman with intrasheath tendon subluxation. **A–C**, Short‐axis US images of the first extensor compartment (orange outlines). In **A**, Doppler demonstrates intrasheath complex fluid and soft tissue with hyperemia, consistent with De Quervain tenosynovitis. In **B**, there is APL (blue outline) and EPB tendinosis (white outline) with a partially visualized longitudinal split tear within the APL tendon (hypoechoic region) and a normal relationship of the APL and EPB tendons. Intrasheath tendon subluxation of the EPB inferior to the APL during dynamic thumb extension maneuvers is seen in **C**. **D** and **E**, Pictures showing the US technique with the transducer positioned in the short‐axis for examination of the first extensor compartment with the hand at rest in **D** and dynamic imaging with active thumb extension in **E**. **F** and **G**, Illustrations of the first extensor compartment normal anatomy (**F**) and with intrasheath subluxation of the EPB inferior to the APL (**G**).

The US demonstrated a mild to moderate amount of slightly complex partially compressible fluid, along with mild non‐compressible soft tissue in the first extensor compartment, which was associated with mild hyperemia on Doppler imaging (Figure 1A). These findings were consistent with right De Quervain tenosynovitis, corresponding to the patient's area of pain and symptoms. There was associated abductor pollicis longus (APL) and extensor pollicis brevis (EPB) tendinosis, with a longitudinal split tear identified within the APL tendon (Figure 1B). Additionally, the US revealed intermittent intrasheath subluxation of the APL and EPB tendons during active dynamic thumb extension maneuvers. This subluxation, which corresponded to the popping sensation and palpable snap described by the patient, is most effectively demonstrated in the annotated static images (Figure 1, B and C) and can also be observed, though to a lesser extent, in online supplemental Video 1. Figure 1, D and E demonstrate dynamic US technique and US transducer positioning for imaging the first extensor compartment while Figure 1, F and G illustrate normal first extensor compartment anatomy and intrasheath subluxation, respectively.

The patient was treated conservatively for this clinically relevant painful popping and locking sensation with oral anti‐inflammatory medication and instructed to follow up after 6 months. However, the patient did not return for follow‐up, and no additional information is available.

### Case 2

A 20‐year‐old ambidextrous man was evaluated in an outpatient orthopedics clinic for an injury sustained to his right fifth digit 3 months prior to presentation. The injury occurred when the finger was caught on an exercise box and hyperextended, though not dislocated. Hand radiographs were negative for fracture or dislocation. The patient reported persistent pain, swelling, and a clicking sensation along the volar aspect of the fifth metacarpophalangeal joint during gripping activities. A dynamic musculoskeletal US was requested for further evaluation.

**Figure 2:**
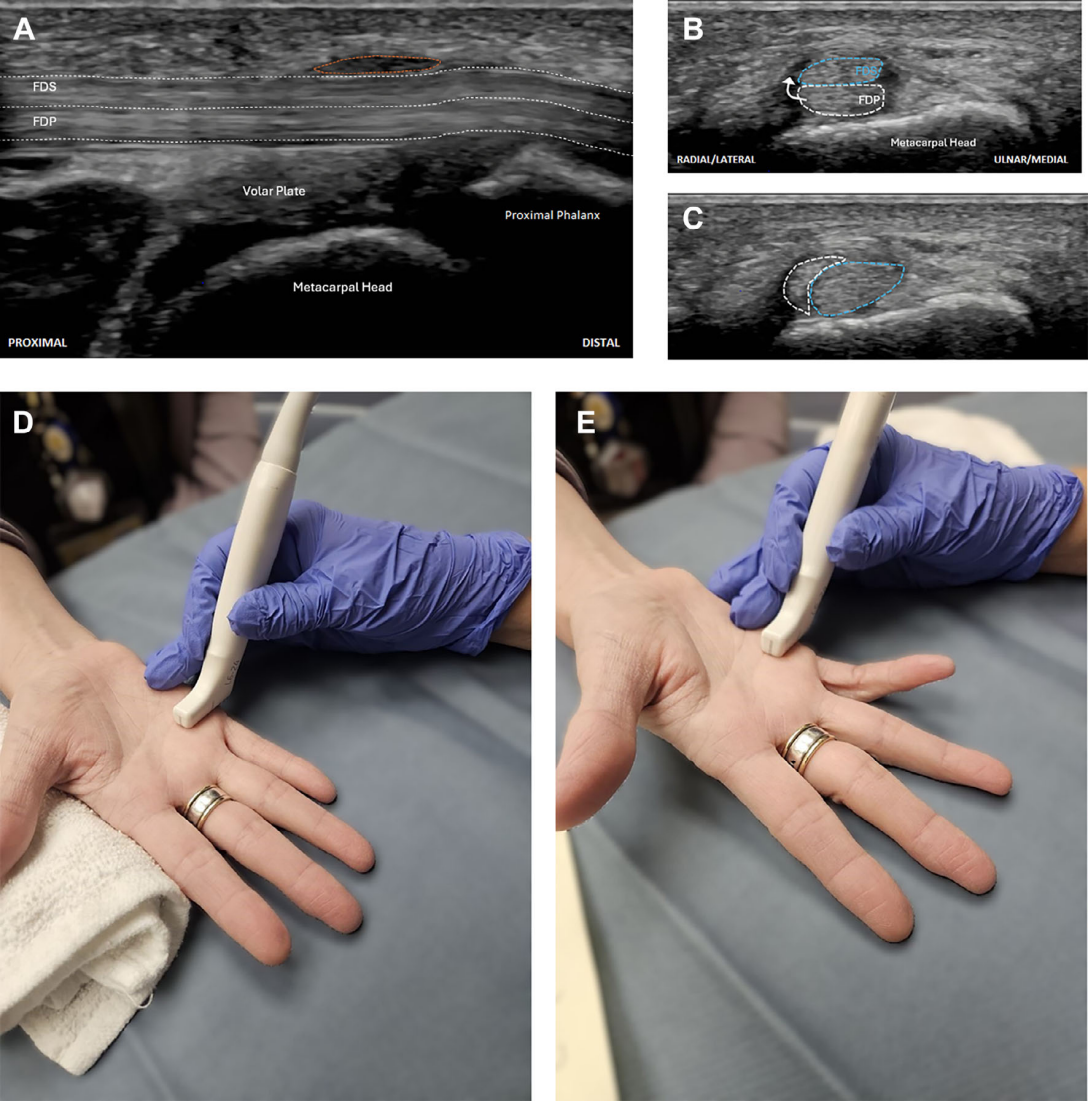
Fifth flexor tendon tenosynovitis and intrasheath subluxation in a 20‐year‐old man. **A**, Long‐axis and **B** and **C**, short‐axis US demonstrate thickening of the A1 pulley (orange outline in **A**) with a normal position of the FDS (blue outline) and FDP (white outline) in **A** and **B**. In **C**, there is intrasheath subluxation during active flexion‐extension maneuvers. **D** and **E**, Pictures showing the US technique with the transducer positioned in the short axis, at the level of the fifth metacarpophalangeal joint/A1 pulley, for examination of the fifth digit flexor tendons at rest in **D** and with active dynamic flexion in **E**.

The US demonstrated thickening of the fifth digit A1 pulley (Figure 2A) and mild non‐compressible hypoechoic distention of the fifth flexor tendon sheath, consistent with mild tenosynovitis. Dynamic US was negative for stenosing tenosynovitis/trigger finger. However, it identified intrasheath subluxation of the flexor digitorum superficialis (FDS) and flexor digitorum profundus (FDP) tendons during active dynamic flexion of the fifth digit (Figure 2, B and C, and online supplemental Video 2). This subluxation correlated to the patient's reported clicking sensation and pain. Figure 2, D and E show the dynamic US technique and transducer positioning for examining the flexor tendons of the fifth digit.

The referring physician advised the patient to avoid activities that could aggravate the painful clicking sensation and suggested conservative treatment. At a 4‐month follow‐up visit to the orthopedic clinic, the patient reported a persistent painful clicking sensation in the fifth digit of the right hand during tight grip activities. Conservative management was recommended again, focusing on avoiding aggravating activities and incorporating range of motion exercises. Surgical intervention was not discussed, and there was no further follow‐up.

### Case 3

A 48‐year‐old woman was seen in an outpatient rheumatology clinic for follow‐up of her history of juvenile idiopathic arthritis, which was managed with hydroxychloroquine. During this visit, she reported an increased prominence of a palpable nodule along the volar aspect of the fourth finger, in the region of the flexor tendons, in addition to bilateral foot symptoms. Her medical history was also relevant for a previous steroid injection for tendinosis at that site. A musculoskeletal US of the bilateral hands and wrists was ordered to evaluate for Dupuytren's contracture/palmar fibromatosis and to assess for subclinical active synovitis given her history.

**Figure 3:**
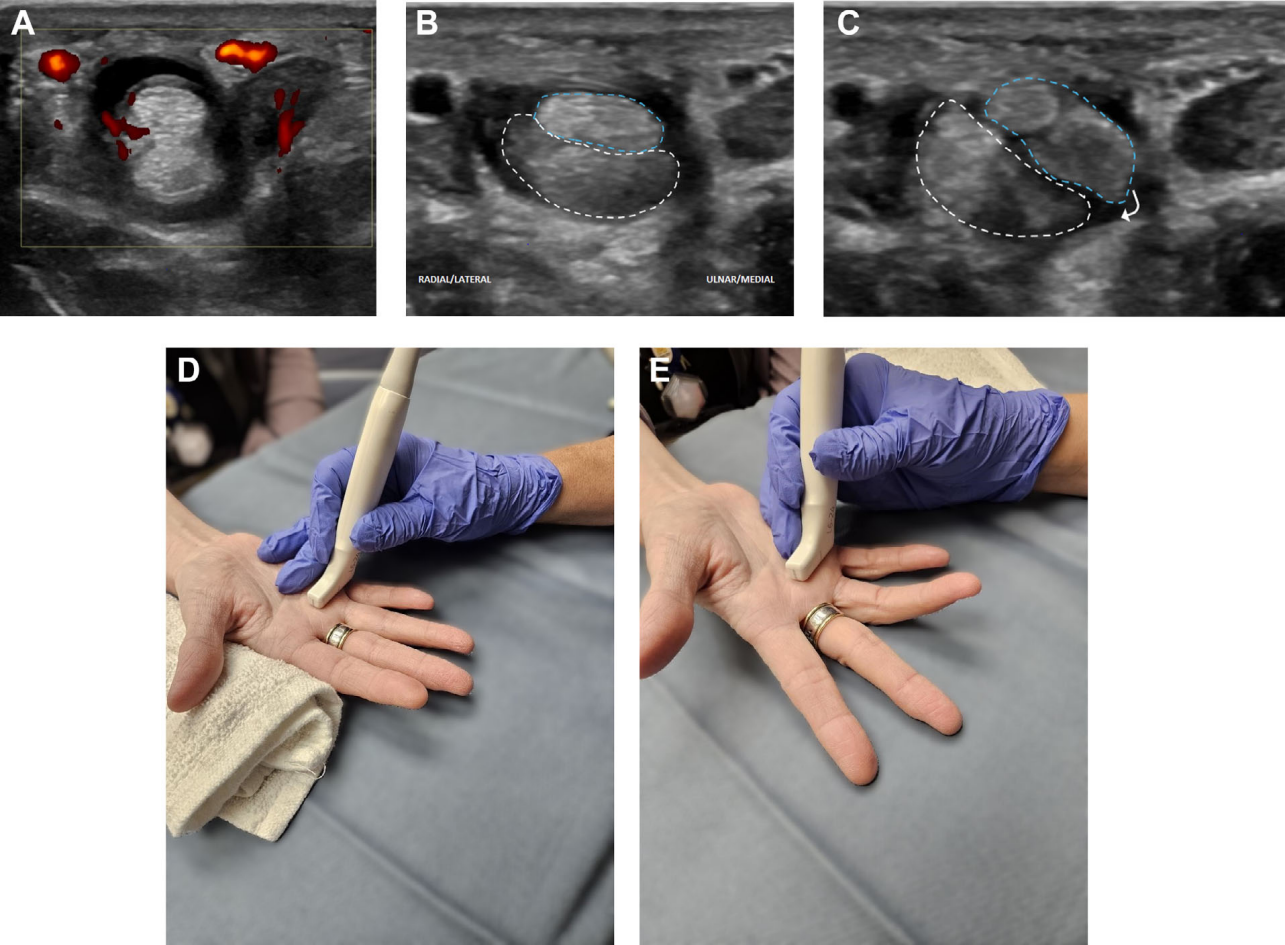
Active flexor tenosynovitis with intrasheath subluxation of the FDS and FDP tendons in a 48‐year‐old woman. **A–C**, Short‐axis images of the fourth digit flexor tendons demonstrating active tenosynovitis with hyperemia on Doppler imaging (**A**). In **B**, there is a normal relationship of the FDS (blue outline) and FDP (white outline) tendons at rest. In **C**, intrasheath subluxation is noted during active dynamic maneuvers. **C–E**, Pictures showing the US technique with the transducer positioned in the short axis, at the level of the fourth metacarpophalangeal joint/A1 pulley, at rest in **D** and with active dynamic flexion in **E**.

The US showed no evidence of active synovitis in the bilateral hand or wrist joints. However, active tenosynovitis was identified in the right fourth flexor tendon sheath, presenting as moderate complex, partially compressible fluid and soft tissue thickening with hyperemia on Doppler imaging (Figure 3A). Furthermore, intrasheath subluxation of the FDS and FDP tendons was noted during active dynamic flexion of the fourth digit (Figure 3, B and C and online supplemental Video 3), corresponding to the region of the palpable abnormality. Although the A1 pulley was thickened, there was free movement of the tendon, indicating no dynamic evidence of stenosing tenosynovitis. Figure 3, D and E show the dynamic US technique and transducer positioning while examining the flexor tendons of the fourth digit.

Given the clinically relevant tenosynovitis and the intrasheath subluxation, a US‐guided steroid injection into the fourth flexor tendon sheath was offered, but the patient opted for more conservative management, including adjustments to her rheumatologic medications. Considering her history of juvenile idiopathic arthritis, the rheumatologist recommended adding sulfasalazine and planned a follow‐up to assess her response. Two months later, she was seen by a hand surgeon who administered a cortisone shot to the fourth digit, which, according to the note, provided immediate relief. Surgical intervention was not discussed.

## Discussion

This unique case review series demonstrates, for the first time to our knowledge, that intrasheath subluxation can occur outside of the ankle, specifically in the hand, and can be a significant source of patient discomfort and disability. The use of musculoskeletal US has significantly increased in recent decades, due to its accessibility, portability, cost‐effectiveness, high spatial resolution, and ability to perform dynamic imaging that correlates with physical examination findings.^5^, ^6^, ^7^, ^8^ Dynamic US is the modality of choice for assessing intrasheath tendon subluxation.^1^, ^2^, ^3^, ^4^

To evaluate tendon subluxation in the hand and wrist using US, begin by selecting the appropriate transducer. Due to the superficial location of most anatomic structures in the hand and wrist, a high‐resolution linear transducer, typically with a frequency of at least 12 MHz, is recommended. Initially, use a transducer with a larger footprint to localize the area of interest. Once identified, switch to a compact, high‐resolution linear transducer, often referred to as a “hockey stick” transducer, if available, with a frequency range of 15–25 MHz. The smaller footprint of this transducer allows for easier maneuverability around compact areas and improves visualization during dynamic maneuvers.

Position the patient seated upright, with their hand placed on an examination table in front of them, either volar surface down or up, depending on the specific area of concern. Rolled towels can be used as necessary to prop up the anatomy of interest. Apply a generous amount of US gel to help maintain contact along small digits and bony surfaces, reducing the risk of losing contact. Apply light pressure to avoid compressing any small amount of fluid present. The tendons should be evaluated in the static position in both the short‐ and long‐axes. Use Doppler imaging on any area of suspected fluid or synovium to assess for hyperemia.

To identify intrasheath subluxation, instruct the patient to actively and slowly flex and extend the affected digit or perform any movement that typically triggers the subluxation while observing the area in the short‐axis (Figures 1, 2, 3). If the patient has difficulty with active movement, the practitioner can assist by passively moving the digit.

**Figure 4:**
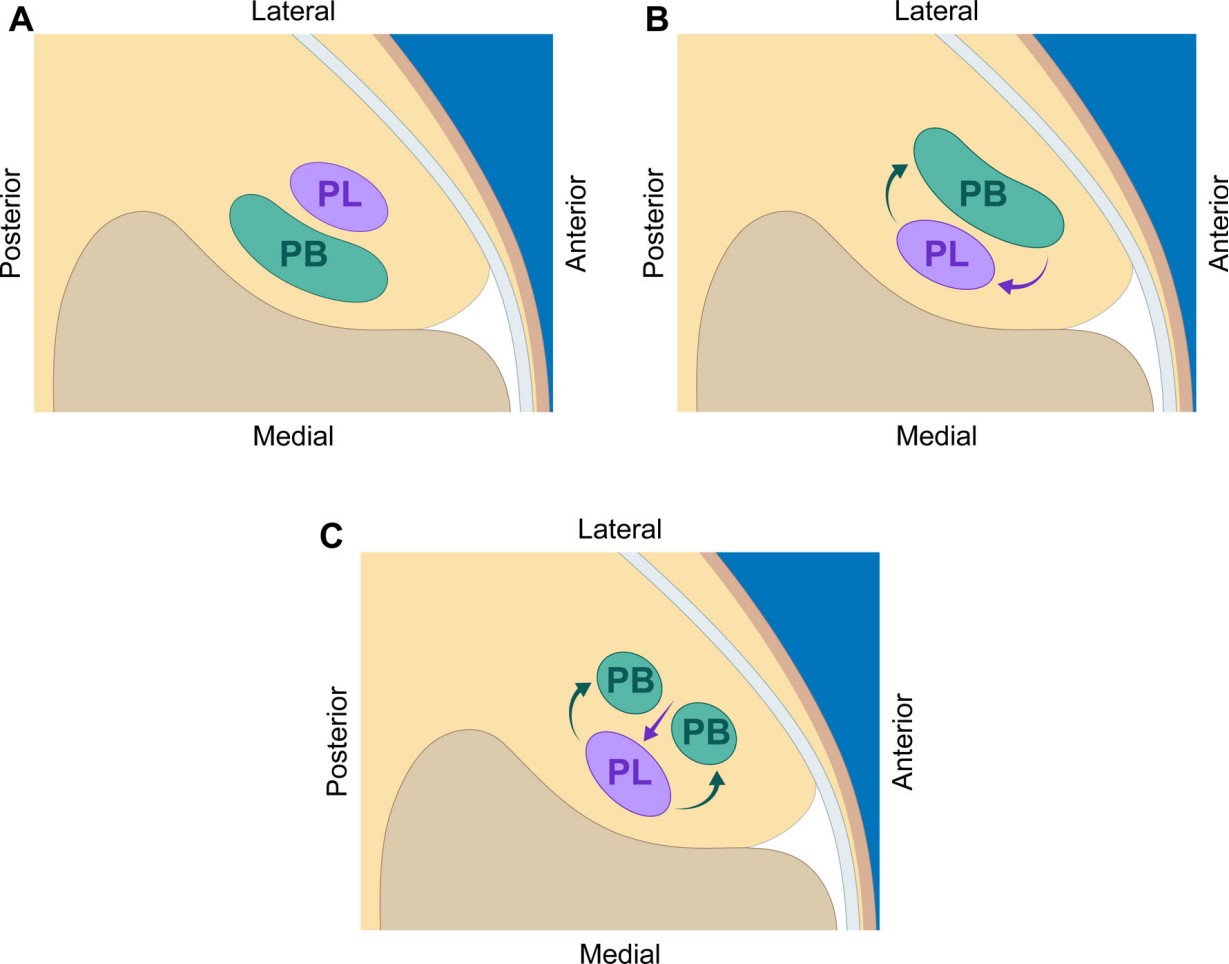
Peroneal tendon anatomy and intrasheath subluxation. **A**, Normal anatomy of the peroneal longus (PL) and peroneus brevis (PB) tendons at the lateral malleolus, with the PL lying superficial to the PB with the overlying retinaculum. **B**, Type A intrasheath subluxation with the PB now moving superficial to the PL tendon. **C**, Type B intrasheath subluxation with the PL subluxing through a longitudinal split tear of the PB tendon.

Until now, peroneal tendon intrasheath subluxation remains the only studied clinical entity in this category. Two types have been described and are shown in Figure 4, B and C. Type A involves the intermittent inversion of the anteroposterior position of intact peroneus longus and brevis tendons, while Type B includes a longitudinal split tear in the peroneus brevis, allowing the longus to sublux through.^1^, ^2^, ^3^, ^4^ These two types can be visualized by dynamically imaging the tendons in the short axis as the patients actively perform dorsiflexion and eversion, circumduction of the ankle, or other ankle movements that reproduce the symptoms (Figure 4).^1^ Initial conservative management may include US‐guided steroid injections of the peroneal tendon sheath.^9^ Persistent or severe cases typically require surgical intervention, traditionally involving open exploration and peroneal groove deepening, though endoscopic methods have also been described.^1^, ^10^

In our first case, we observed De Quervain tenosynovitis with intrasheath subluxation of the APL and EPB tendons. The second and third cases demonstrated flexor tenosynovitis with thickening of the A1 annular pulley and intrasheath subluxation of the FDS and FDP tendons. All three cases would be similar to Type A described above. Inflammation or thickening of the tendon sheath may impede smooth tendon movement, potentially leading to subluxation.

An important normal anatomic variant to be aware of is a septum within the first extensor compartment, located between the APL and EPB tendons, dividing it into two subcompartments. The incidence of the septum is reported to be ~50–77.5%. Furthermore, even in the absence of a sonographically visible septum, it has been shown that the US detection of a small subjacent osseous ridge at the level of the distal radius is an indirect sign of the presence of this septum. If present, this septum would be expected to prevent intrasheath tendon subluxation in the first extensor compartment. Furthermore, if the septum or osseous ridge is present and an US‐guided first extensor compartment tendon sheath injection is planned, either subcompartment or both must be targeted for injection.^11^, ^12^, ^13^, ^14^, ^15^

An additional important normal anatomic variant of the first extensor compartment that clinicians should be aware of is the presence of multiple slips of the APL tendon and, occasionally, the EPB tendon. It is crucial not to mistake these for longitudinal split tears, partial‐thickness tearing, or fissuring. A characteristic “lotus root” appearance of these multiple slips involving the APL tendon can occur in ~95% of cases. Recognizing these normal variations is essential to avoid misdiagnosis and ensure appropriate treatment.^15^

**Figure 5:**
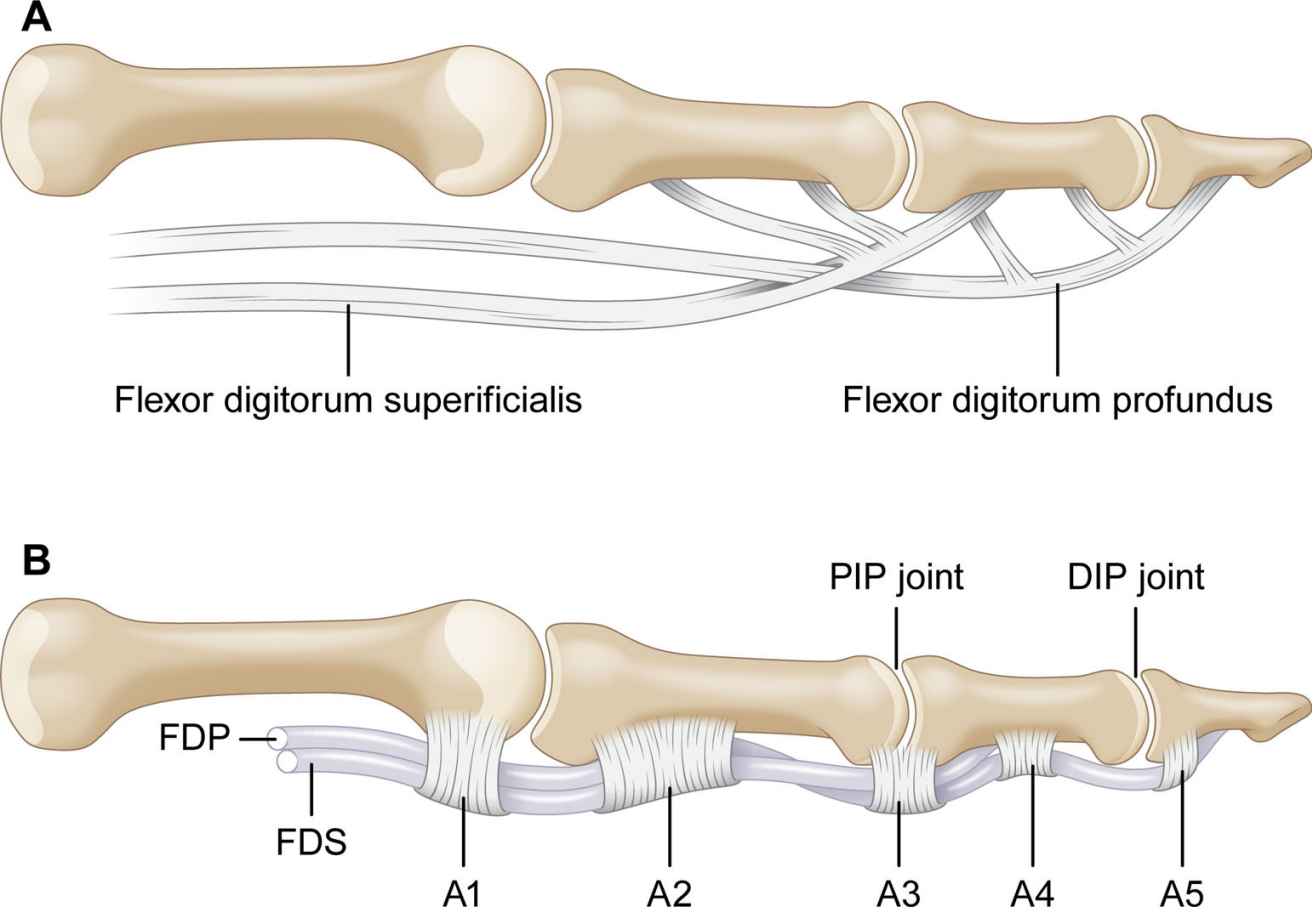
Normal flexor tendon anatomy of the hand. **A**, Normal position of the flexor digitorum superficialis (FDS) and flexor digitorum profundus (FDP) tendons. From the metacarpophalangeal joint to the mid aspect of the proximal phalanx, the FDS lies superficial to the FDP. At the mid aspect of the proximal phalanx, the FDS divides to surround the FDP. Its two slips then reunite deep to the FDP at the proximal aspect of the middle phalanx. **B**, The annular and pulleys along the volar aspect of the fingers that cover the FDS and FDP tendons, retaining the flexor tendons against bone.

An understanding of the digital flexor system is also key to exploring potential mechanisms of intrasheath subluxation in the hand, particularly seen in the second and third cases (Figure 5A). The second through fifth digits of the hand each have two flexor tendons, the FDS and FDP tendons. From the metacarpophalangeal joint to the middle aspect of the proximal phalanx, the FDS tendon lies superficial to the FDP tendon. At the mid aspect of the proximal phalanx, the FDS tendon divides to surround the FDP tendon. Its two tendon slips then reunite deep to the FDP tendon at the proximal aspect of the middle phalanx via an X‐shaped connection termed the chiasma crurale, before inserting on both sides of the middle phalanx. The FDP tendon, now superficial to the FDS tendon, continues distally through the opening created by the two FDS tendon slips and inserts on the volar aspect of the base of the distal phalanx.^16^

The annular and cruciform pulleys form a fibrous tendon sheath on the volar aspect of the fingers that overlie the FDS and FDP tendons (Figure 5B). While the cruciform pulleys are more flexible and allow flexion of the interphalangeal joints, the thicker annular pulleys retain the flexor tendons against the bone during finger flexion, preventing bowstringing.^15^ Partial injury to and resultant laxity of the annular pulleys could then allow for intrasheath subluxation of the FDS and FDP tendons. We hypothesize that potentially tenosynovitis combined with a partial injury of the annular pulley may result in intrasheath tendon subluxation in the hand. However, further investigations with a larger cohort and intraoperative findings would be needed to validate this theory.

## Conclusion

While traditionally thought to occur only in the ankle, we have identified intrasheath subluxation in the hand. Knowledge of this condition and its sonographic features is crucial in order to recognize this important clinical and diagnostic challenge. As musculoskeletal US use continues to increase, particularly with dynamic imaging, hand intrasheath tendon subluxation may be encountered more frequently in clinical practice. Further research is needed to determine the exact causes and sonographic characteristics of hand intrasheath subluxation, which could help refine diagnostic and treatment strategies for patients.

## Ethics Statement

All procedures performed in studies involving human participants were in accordance with the ethical standards of the institutional and/or national research committee and with the 1964 Declaration of Helsinki and its later amendments or comparable ethical standards. Informed consent was waived.

## Supporting information


**Supplemental Video 1.** Short‐axis cine of the wrist first extensor compartment demonstrating intrasheath subluxation of the EPB under the APL during dynamic thumb extension maneuvers.

**Supplemental Video 2.** Short‐axis cine of the fifth digit flexor tendons demonstrating intrasheath subluxation of the FDS and FDP tendons during active dynamic flexion and extension maneuvers.

**Supplemental Video 3.** Short‐axis cine of the fourth digit flexor tendons demonstrating intrasheath subluxation of the FDS and FDP tendons during dynamic maneuvers.

**Supplemental Data S1.** Video Placeholder Image Captions: Video 1. Short‐axis image of the wrist's first extensor compartment demonstrating intrasheath subluxation of the EPB under the APL during dynamic thumb extension maneuvers.
Video 2. Short‐axis image of the fifth digit flexor tendons demonstrating intrasheath subluxation of the FDS and FDP tendons during active dynamic flexion and extension maneuvers.
Video 3. Short‐axis image of the fourth digit flexor tendons demonstrating intrasheath subluxation of the FDS and FDP tendons during dynamic maneuvers.

## Data Availability

Data sharing not applicable to this article as no datasets were generated or analysed during the current study.
