# Supplementary figures and images for: Intrasheath Tendon Subluxation Beyond the Ankle: The First Case Series in the Hand
Source: J Ultrasound Med. 2025 Jul 31;44(12):2361–9. doi: 10.1002/jum.70020 (PMC12611448; doi:10.1002/jum.70020)

Embedded Video Placeholder Images

Video 1 – 0:00:03
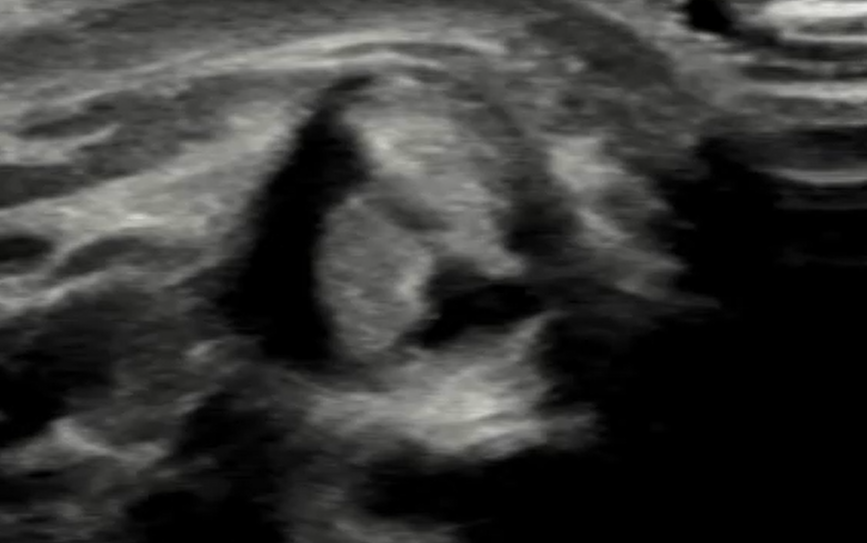


Video 2 – 0:00:06


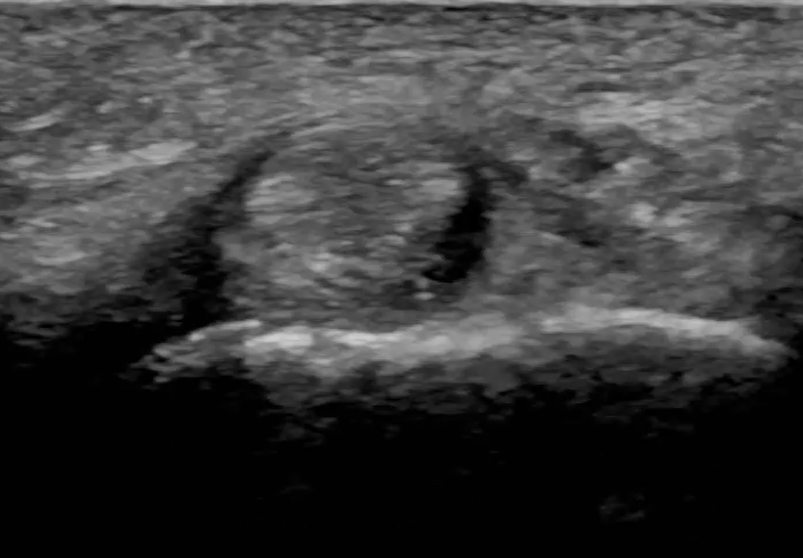


Video 3 – 0:00:03


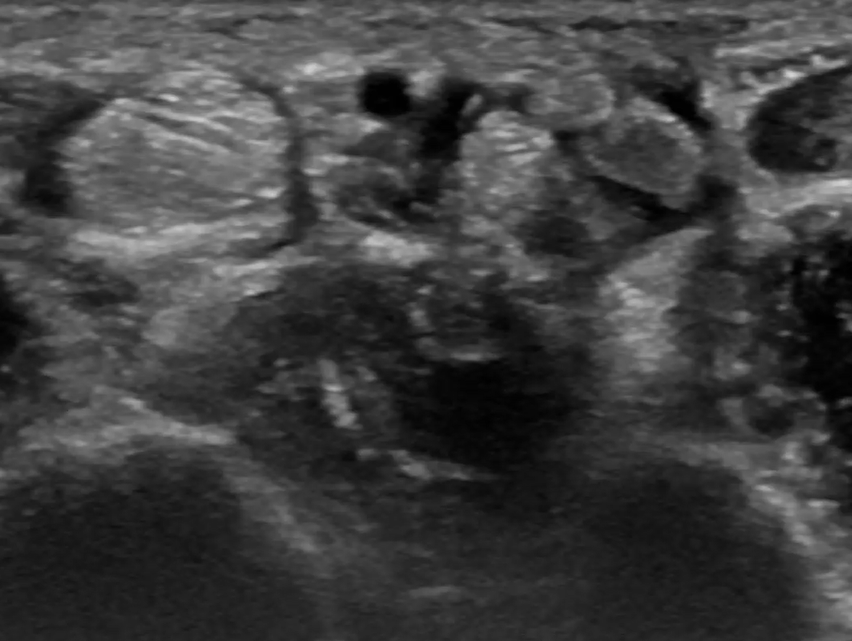

Supplement: Supplementary file 4 — Supplemental Data S1. Video Placeholder Image Captions: Video 1. Short‐axis image of the wrist's first extensor compartment demonstrating intrasheath subluxation of the EPB under the APL during dynamic thumb extension maneuvers. Video 2. Short‐axis image of the fifth digit flexor tendons demonstrating intrasheath subluxation of the FDS and FDP tendons during active dynamic flexion and extension maneuvers. Video 3. Short‐axis image of the fourth digit flexor tendons demonstrating intrasheath subluxation of the FDS and FDP tendons during dynamic maneuvers. [file JUM-44-2361-s001.docx]
